# Standardization of Body Composition Status in Patients with Advanced Urothelial Tumors: The Role of a CT-Based AI-Powered Software for the Assessment of Sarcopenia and Patient Outcome Correlation

**DOI:** 10.3390/cancers15112968

**Published:** 2023-05-29

**Authors:** Antonella Borrelli, Martina Pecoraro, Francesco Del Giudice, Leonardo Cristofani, Emanuele Messina, Ailin Dehghanpour, Nicholas Landini, Michela Roberto, Stefano Perotti, Maurizio Muscaritoli, Daniele Santini, Carlo Catalano, Valeria Panebianco

**Affiliations:** 1 Department of Radiological Sciences, Oncology and Pathology, Sapienza University of Rome, 00161 Rome, Italy; 2 Department of Maternal Infant and Urologic Sciences, Sapienza University of Rome, 00161 Rome, Italy; 3 Department of Translational and Precision Medicine, Sapienza University of Rome, 00161 Rome, Italy

**Keywords:** Artificial Intelligence software, Sarcopenia, Computed Tomography, Urogenital Tumors, Oncology

## Abstract

**Simple Summary:**

Artificial Intelligence (AI)-driven software that utilizes Computed Tomography (CT)images has the capability to automatically assess body composition and diagnose sarcopenia. Our research indicates that combining standardized CT staging methods with sarcopenia analysis could assist in identifying patients with advanced urothelial tumors who may benefit from customized nutritional therapies, ultimately resulting in improved outcomes and quality of life. The AI tool can represent a means to increase the clinical value of CT imaging reports and to promote the development of precision medicine.

**Abstract:**

Background: Sarcopenia is a well know prognostic factor in oncology, influencing patients’ quality of life and survival. We aimed to investigate the role of sarcopenia, assessed by a Computed Tomography (CT)-based artificial intelligence (AI)-powered-software, as a predictor of objective clinical benefit in advanced urothelial tumors and its correlations with oncological outcomes. Methods: We retrospectively searched patients with advanced urothelial tumors, treated with systemic platinum-based chemotherapy and an available total body CT, performed before and after therapy. An AI-powered software was applied to CT to obtain the Skeletal Muscle Index (SMI-L3), derived from the area of the psoas, long spine, and abdominal muscles, at the level of L3 on CT axial images. Logistic and Cox-regression modeling was implemented to explore the association of sarcopenic status and anthropometric features to the clinical benefit rate and survival endpoints. Results: 97 patients were included, 66 with bladder cancer and 31 with upper-tract urothelial carcinoma. Clinical benefit outcomes showed a linear positive association with all the observed body composition variables variations. The chances of not experiencing disease progression were positively associated with ∆_SMI-L3, ∆_psoas, and ∆_long spine muscle when they ranged from ~10–20% up to ~45–55%. Greater survival chances were matched by patients achieving a wider ∆_SMI-L3, ∆_abdominal and ∆_long spine muscle. Conclusions: A CT-based AI-powered software body composition and sarcopenia analysis provide prognostic assessments for objective clinical benefits and oncological outcomes.

## 1. Introduction

Cancer cachexia is a complex condition where there is a progressive reduction in skeletal muscle mass that cannot be fully restored by traditional nutritional intervention, resulting in a gradual decline in bodily functions. The loss of muscle mass and malnutrition are common conditions found in cancer patients, with an incidence ranging from 25% to 60% depending on the type of cancer, stage of disease, and type of treatment [1]. The low muscle mass represents one of the criteria for malnutrition diagnosis according to Global Leadership Initiative on Malnutrition (GLIM) guidelines [2]. Despite affecting about half of patients at diagnosis as described in the PreMIO observational study conducted on over 1000 patients suffering from different solid tumors [3], malnutrition is often underestimated and not fully considered by urogenital oncologists. Standardization of the sarcopenia definition is an urgent issue in oncology [4] indeed no screening tests to assess sarcopenia and/or the risk of malnutrition are usually requested at disease diagnosis. Cachexia has been shown to be a significant poor prognostic factor for relapse-free survival of patients affected with urothelial tumors. [5] Currently, there is no standard method for diagnosing sarcopenia in genitourinary tumor [6] patients, particularly for urothelial tumors, [7] differently from other diagnostic procedures [8,9,10,11,12,13]. A Computed Tomography (CT)-based imaging for the assessment of muscle mass is a very accurate tool for the detection of sarcopenia in oncological patients. However, the need of tracing manually the muscle groups to calculate body composition parameters is a costly and time-consuming limitation [14]. On the other hand, the development and progressive implementation of a dedicated CT imaging-based artificial intelligence (AI)-powered software has allowed for automated quantification of muscle mass by assessing skeletal muscle cross-sectional area (SMA) at the level of the third lumbar vertebra (L3), providing an ease calculation of another key body composition variable, the skeletal muscle index (SMI) obtained by normalizing the SMA to the patient’s height (m^2^) [15]. Indeed, AI gives an opportunity to automate the process of sarcopenia assessment [16], providing meaningful clinical measurements that can be considered independent imaging biomarkers for overall survival, as it is carried out for other tumors [17]. Based on the hypothesis that sarcopenia is associated with poorer patients’ oncological outcomes, the objective of the study was to verify if sarcopenia, evaluated using a CT-based AI-powered software, can predict objective clinical benefit in terms of tumor response rate to systemic chemotherapy, for advanced urothelial tumors. Additionally, the study aimed, as a secondary endpoint, to establish a correlation between sarcopenia and cancer outcomes.

## 2. Materials and Methods

### 2.1. Study Design

This was a retrospective single-center observational study that received formal approval from the Institutional Review Board, with a waiver of informed consent. The study was conducted in accordance with the guidelines for good clinical practice with ethical principles as reported in the latest version of the Declaration of Helsinki.

The medical records data were collected for 97 patients with a histologically confirmed diagnosis of urothelial tumors before the initiation and after 4–6 cycles of chemotherapy (at the first oncological reassessment) from January 2018 to January 2021 at our institution. The data collected included gender, age, height, weight, body mass index, number of drugs taken (not related to cancer treatment), ECOG Performance Status (PS), and clinical- and radiological stages.

Inclusion criteria were the following: age > 18 years, diagnosis of advanced urothelial tumor, availability of CT scan before and after treatment, and follow-up of at least 60 months.

Exclusion criteria were as follows: Patients with a life expectancy lower than 3 months or affected by any chronic inflammatory pathology in active status, with no long-term clinical information available, unsuitable for chemotherapy treatment, or with any contraindication to perform CT examinations.

As per institutional protocols, the staging CT scans were performed at both baselines and after 6 months of treatment.

### 2.2. Image Acquisition and Analysis

Images were acquired on a multidetector CT scanner (Somatom Sensation 64; Siemens Healthineers, Erlangen, Germany). Scanning parameters were as follows: tube voltage, 120 kVp; tube current, 100–250 mAs; pitch, 1.2; and collimation, 0.625–0.75 mm. Images were reconstructed using a 1-mm slice thickness on axial, coronal, and sagittal planes, using both soft tissue kernel (B31f) and lung kernel (B75f) reconstruction.

The CT acquisition protocol used was the standard for urothelial tumors and it included: A pre-contrast phase, a corticomedullary phase (data acquisition 25–35 s after contrast media injection); a nephrographic phase (80–100 s); an excretory phase (10–16 min).

**Figure 1:**
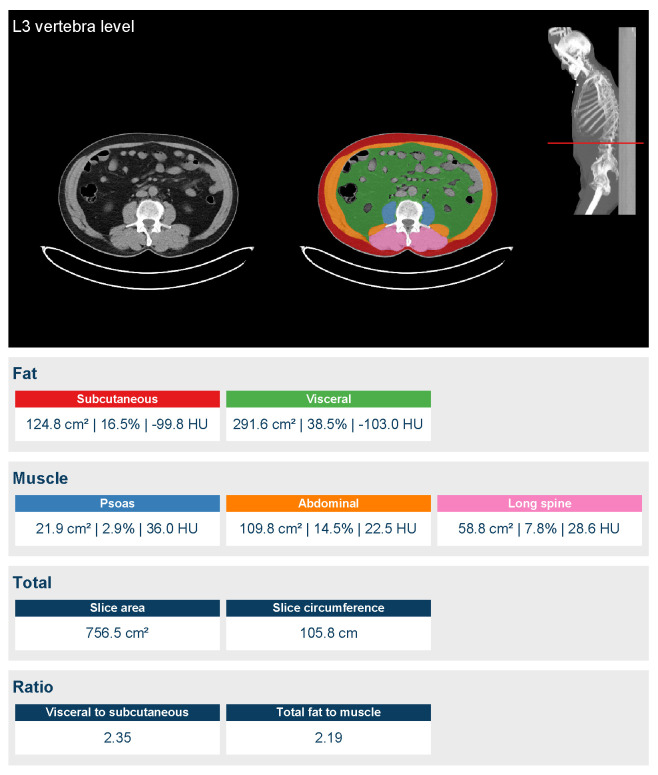
Case example of the automatic segmentation performed by the software at the level of the third lumbar vertebra.

The Quantib body composition^®^ software (Rotterdam, Netherlands) was used to measure body composition quantitatively [18]. This software analyzed CT images of patients taken during staging CT examinations stored in our institutional picture archiving and communication system (PACS) selecting just the non-contrast phase. The software focused on the L3 vertebral body level and automatically segmented the images to determine the areas of the abdominal, psoas, and long spine muscles. Finally, the software generated in a few minutes a form displaying the relevant values, as shown in Figure 1.

Manual corrections were not made, and patients with grossly incorrect segmentations were excluded from the study. There was no specific definition for what constituted an incorrect segmentation; all segmentations were considered either of high quality or had only a small portion of the muscle volume accurately delineated. Minor errors were permitted without any additional correction.

### 2.3. Sarcopenia and Response to Therapy Definition

The SMA was obtained by summing the muscle areas of the psoas muscle, abdominal muscle, and long spine muscle.

The SMI was obtained by normalizing the SMA by the patients’ squared height. According to the literature, an SMI cut-off value for the sarcopenia definition was set at <55 cm^2^/m^2^ for men and <39 cm^2^/m^2^ for women [19]. The body mass index (BMI) was calculated as generally obtained (weight/height^2^) before and after therapy.

The RECIST 1.1 criteria [20] were used to identify and classify patients’ responses to therapy. Complete response (CR) was defined as the disappearance of all target lesions for a period of at least one month; partial response (PR) as at least a 30% decrease in the sum of the maximum diameter of target lesions, taking as reference the baseline sum of the longest diameter (LD); stable disease (SD) as neither sufficient shrinkage to qualify for PR nor sufficient increase to qualify for PD, taking as reference the smallest sum LD since the treatment started; progressive disease (PD) as at least a 20% increase in the sum of the LD of target lesions, taking as reference the smallest sum LD recorded since the treatment started or the appearance of one or more new lesions. Overall survival (OS) was defined as the time between diagnosis and last contact or date of death. Clinical benefit rate (CBR) was defined as the percentage of advanced-stage patients who achieve complete response, partial response, or at least six months of stable disease as a result of therapy [21].

### 2.4. Statistical Analysis

Statistical analyses along with reporting and interpretation of the results were conducted according to the previously described methodology [22] and consisted of four separate analytical steps [23,24].

Initially, descriptive statistics were used to summarize pertinent study information. The association between sarcopenia and clinical variables was tested by Fisher’s exact test or Mann–Whitney U test.

Second, a set of regression analyses was performed to assess the initial degree of correlation between CT scan AI-based Quantib Body Composition^®^ SMI-L3 and each single anthropomorphic sarcopenia-related measures (i.e., CT-defined subcutaneous and visceral fat as well as psoas, abdominal and long spine muscle). This was tested both at baseline and repeated with the same data after the first cycle of systemic chemotherapy administration. Moreover, a multivariable linear regression model was developed including those clinic-demographic and tumor-related features commonly associated with clinical and survival outcomes (i.e., age at diagnosis, gender, ECOG performance status, number of medications taken unrelated to cancer therapy, as well as tumor location and stage) in order to identify which association was more significantly correlated with the computed SMI-L3 and the sarcopenia pre-established cut-off criteria [25].

Third, the clinical benefit outcome measured at the end of the systemic therapy was defined by the presence of partial/complete radiological response (RaR) as well as the confirmation of the stable disease in contrast with the documented progression of advanced urothelial carcinomas. Given the known association of sarcopenia status with male or female gender, a set of bivariable logistic regression was modeled between each computed sarcopenia-related feature adjusted for gender status and the dichotomized clinical benefit outcome. Additionally, a multivariable logistic model by clinical and demographic confounders was further performed to identify sarcopenia-related predictors independently associated with clinical benefit endpoint.

As the fourth analytic step, we investigated the association of SMI-L3 both as a continuous and dichotomized covariate with OS using univariable Cox regression analysis. Univariable survival estimates were plotted using the Kaplan–Meier method. The log-rank test was used to assess the difference in OS between sub-groups. A multivariate Cox proportional hazards model was also developed by adjusting for previously mentioned confounders also associated with survival outcomes. Finally, sarcopenia status variation as a function SMI-L3 variation (∆_SMI-L3) as well as any AI-computed subcutaneous/visceral fat or muscular anthropometric variations were forced, using locally weighted scatter plot smoother (LOWESS) function, against the multivariable-adjusted predicted probability models for clinical benefit and survival assessment. This was meant to graphically depict the influence of body composition variations on the pre-established endpoints at the moment of primary disease diagnosis. Statistical analysis was performed using Stata version 17.1 (Stata Corporation, College Station, TX, USA) with statistical significance set as *p* < 0.05.

## 3. Results

### 3.1. Demographic, Tumor- and Sarcopenia-Related Characteristics of the Study Population

**Figure 2:**
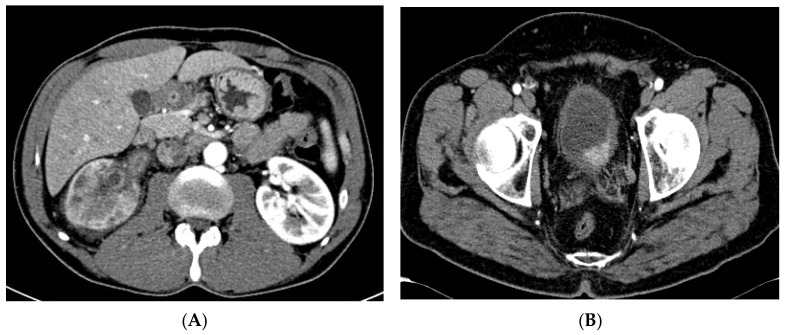
(**A**) Computed tomography (CT) image of a 75-year-old men with advanced right Upper Tract Urothelial Tumor (SMI value = 40.54 cm^2^/m^2^; (**B**) CT image of 72-year-old men with advanced Bladder Tumor on left posterior wall. (SMI value = 44.86 cm^2^/m^2^).

We retrospectively reviewed the medical records of 97 patients with a histologically confirmed diagnosis of an advanced urothelial tumor of the upper urinary tract (33; 34.1%) (Figure 2) and urothelial tumor of the urinary bladder (64; 65.9%) (Figure 2) [26].

The median age of non-sarcopenic vs. sarcopenic patients was 73 (Interquartile range [IQR], 64–76) vs. 69 (IQR, 64–74) before and 73 (IQR, 66–76) vs. 68 years (IQR, 64–74) after therapy.

There were no differences in the distribution of the clinical and demographical factors among the non-sarcopenic and sarcopenic patients both at baseline and after therapy.

The SMI-L3 variable (cm^2^/m^2^), as well as the additional variables, were significantly different before and after therapy (*p* < 0.001) (Table 1).

### 3.2. Correlation between AI Skeletal Muscle Index (SMI-L3) and Anthropomorphic Sarcopenia-Related Variables Pre-/Post-Systemic Treatment

At baseline, out of 97 patients, 34 (66.7%) males and 17 (33.3%) females met the definition criteria calibrated on the CT-defined AI software SMI-L3 and were considered as affected by sarcopenic status.

At univariable linear regression modeling, we found an increasingly positive and constant association between SMI-L3 and each anthropometric sarcopenia-related feature. The coefficient correlation of determination (r^2^) was especially relevant when assessing SMI-L3 and the abdominal muscle area (r^2^: 0.726, d.f.: 95, value: 0.450, 95% CI: 0.394–0.507, *p* < 0.0001), followed by the long spine and psoas area (r^2^: 0.599, d.f.: 95, value: 0.740, 95% CI: 0.617–0.863, *p* < 0.0001 and r^2^: 0.463, d.f.: 95, value: 1.471, 95% CI:1.149 –1.794, *p* < 0.0001). At multivariable linear regression, the goodness of fit statistics for the SMI-L3 model reached the highest degree of correlation (r^2^: 0.857) and the correlations were confirmed with the visceral fat area as well as with the psoas, long spine, and abdominal muscle area (cm^2^/m^2^) independently from demographic and clinical confounders (Figure S1A,B).

After the first cycle of systemic therapy administration, the observed correlations remained stable and highly significant at both unilinear regression with the muscle-skeletal components (i.e., abdominal, psoas, and long spine muscle area [cm^2^/m^2^]) reaching the highest degree of correlation with SMI-L3 also when adjusted with clinic confounders (r^2^: 0.874) (Figure 2A,B).

### 3.3. Baseline and Early Predictors of Clinical Benefit Measured at the Completion of Systemic Therapy

**Figure 3:**
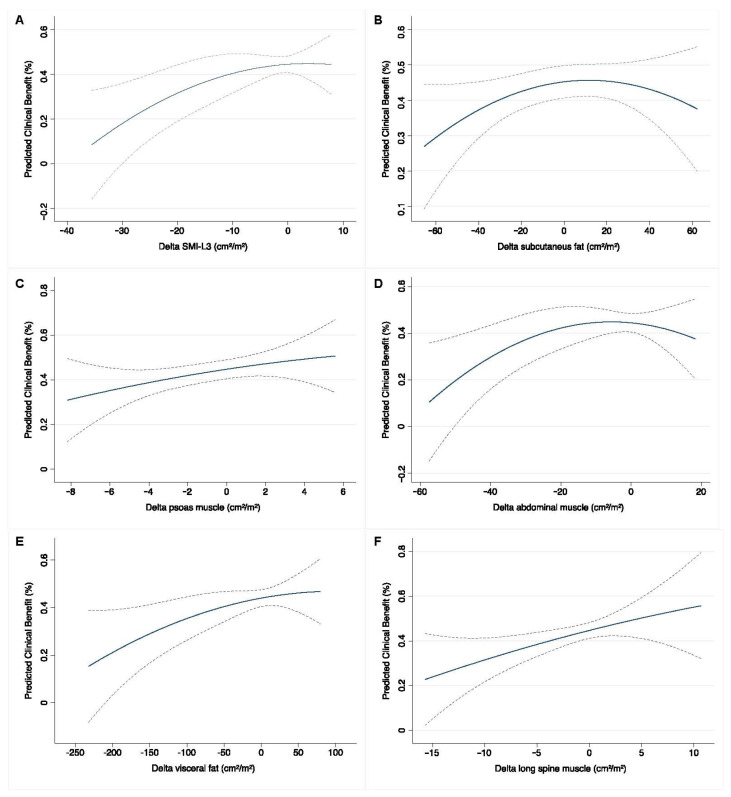
LOWESS functions depicting the predicted probability for RaR clinical benefit outcomes (**A**–**F**).

In total, all patients received a median number of 5 cycles of platinum-based chemotherapy which was mainly represented by Gemcitabine plus Cisplatin (GC) (53, 54.6%) or Carboplatin (44, 45.4%) for patients with renal insufficiency or frailty. The CT scan performed after 4–6 cycles, at the first oncological reassessment, revealed that the overall number of subjects with SMI-L3 defined sarcopenia status was 52 (53.6%). Among these, sarcopenic patients who exhibited disease progression were 26 (50%) while the remaining was associated with both complete/partial RaR (26, 50%). Among the non-sarcopenic group, after chemotherapy, 16 (35.6%) had progression of disease while 29 (64.4%) had complete/partial response. At univariable logistic regression adjusted by gender, baseline predictors for clinical benefit outcomes were represented by the sole abdominal, psoas muscle area, and subcutaneous fat area (aOR: 0.97, 95% CI: 0.95–0.99, aOR: 0.90, 95% CI: 0.82–0.99 and aOR: 0.99, 95% CI: 0.98–1, respectively). However, although not statistically significant, both SMI-L3 and its derived sarcopenia-related cut-off demonstrated an overlapping trend toward significance in line with the other individual aforementioned predictors (aOR: 0.96, 95% CI: 0.92–1.1 and aOR: 2.33, 95% CI: 0.99–5.52) (Table S1A). Notably, at this baseline assessment, none of these anthropometric features resulted independently able to induce relevant clinical benefit outcomes except from some expected clinical variables. Interestingly, after the first oncological reassessment of disease sarcopenic status both by SMI-L3 coefficient and its standardized cut-off, was found independently predicting clinical benefits (OR: 0.93, 95% CI: 0.88–0.98 and OR: 2.31, 95% CI: 1.15–5.78) (Table S1B). Finally, the trajectory of the LOWESS functions depicting the predicted probability for RaR clinical benefit outcomes showed an almost linear positive association with all the observed body composition variables variations between pre-/post-systemic treatment. (Figure 3). This was especially true for ∆_SMI-L3 (Figure 3A) and ∆_psoas (Figure 3C) and ∆_long spine muscle area (cm^2^/m^2^) (Figure 3F) where the chances of not experiencing disease progression increased from the ~10–20% up to ~45–55%. 

### 3.4. Baseline and Early Determinates for Overall Survival

**Figure 4:**
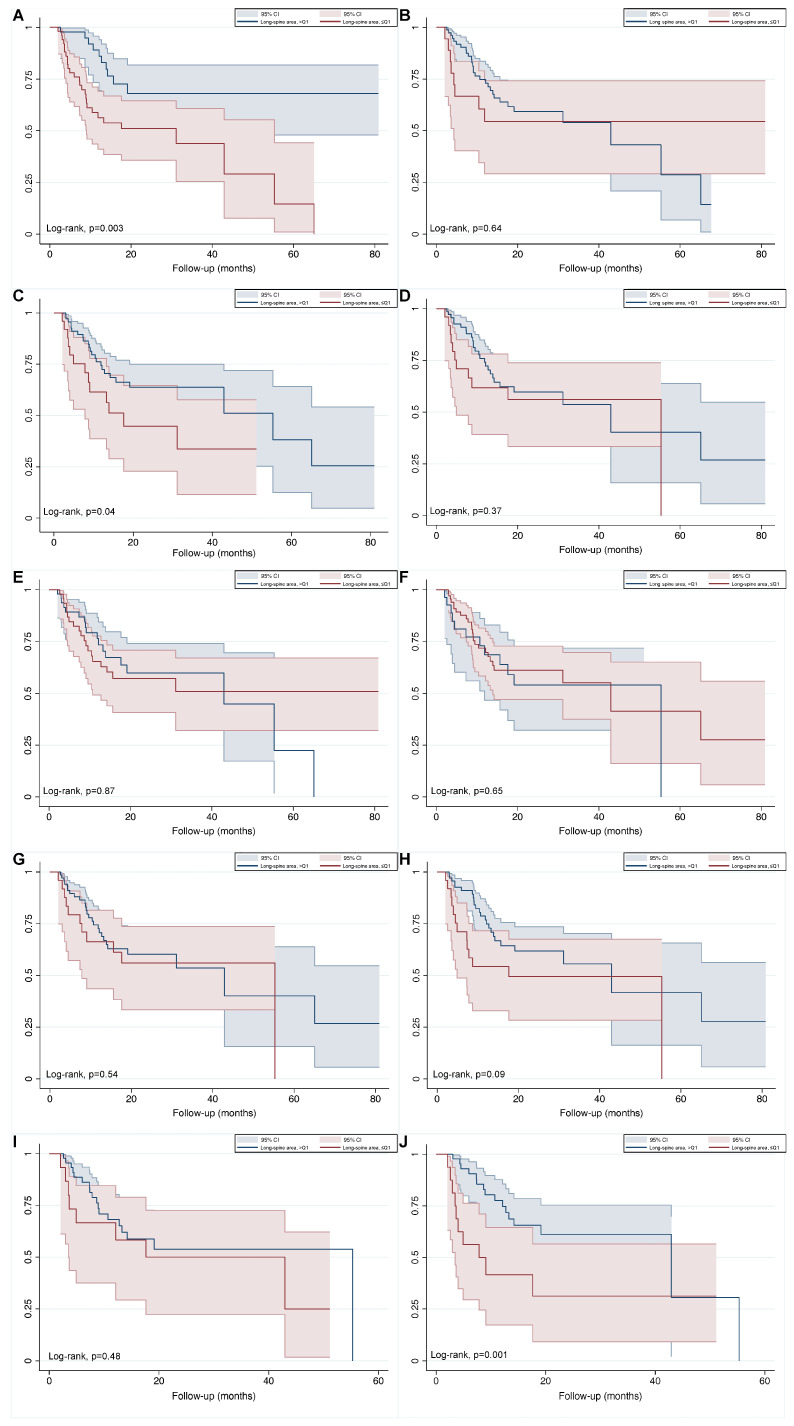
The effect of sarcopenia on OS depicted by Kaplan-Meier plots (**A**–**J**).

Within a median follow-up time of 17.43 months (IQR 1.6–80.9), months, 37 (38.1%) subjects were deceased by any cause. In the sarcopenic cohort, 28 patients (75.8%) were recorded as having passed away, while 9 patients (24.3%) were recognized as survivors. SMI-L3 variation (∆-SMI-L3) was significantly discordant across the two sub-groups ranging from a median value of −1.857 (standard deviation [SD] 5.784), respectively. The univariable effect of sarcopenia and other subgroups on OS has been depicted in Kaplan–Meier plots shown in Figure 4.

As expected, at univariable Cox regression modeling before and after chemotherapy, higher registered values of sarcopenic and anthropometric measures had been associated with reduced OS. This was also independently true at multivariable assessment for SMI-L3 and the subsequent sarcopenia definition at baseline (HR: 0.95, 95% CI: 0.92–0.99 and HR: 3.80, 95% CI: 1.72–8.41) (Table S2A). and after the first cycle of therapy (HR: 0.94, 95% CI: 0.91–0.98 and HR: 3.29, 95% CI: 1.51–7.17) (Table S2B).

**Figure 5:**
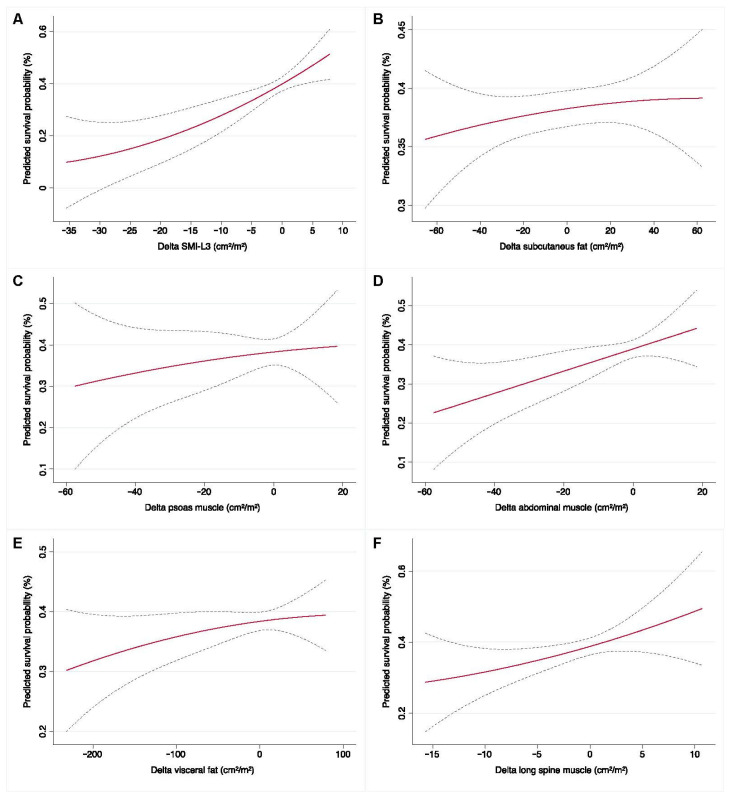
LOWESS function to model ∆-SMI-L3 for the predicted survival probability derived from multivariable Cox regression model (**A**–**F**).

Moreover, when implementing the LOWESS function to model ∆-SMI-L3 on the predicted survival probability derived from the multivariable Cox regression model (Figure 5), greater survival chances were matched by those patients achieving wider ∆_SMI-L3 over the course of follow-up (10–20% vs. 50–60%) (Figure 5A). This was noted especially for ∆_abdominal (Figure 5D) and long spine muscle area (Figure 5F) (cm^2^/m^2^) which varied from ~20–30% up to ~50–55% when a consistent muscular mass was gained. 

## 4. Discussion

Accurate prediction of individual cancer patient’s response to chemotherapy remains a goal in the field of oncology.

The development of sarcopenia is a result of tumor progression and systemic inflammation caused by the tumor, so its presence indicates tumor aggressiveness. In addition, sarcopenic patients are characterized by poor general health and physical performance, which can contribute to a worse prognosis for cancer-bearing patients. The effectiveness of sarcopenia as a prognostic biomarker can be attributed to its distinctive hybrid nature [27].

Several studies have investigated the association between sarcopenia, assessed by AI tools and oncological outcomes, [28]; including patients with breast, [29] gastric [30], endometrial [31] and cervical cancer [32]. Focusing on genitourinary tumors [33], Wu et al. (2019) [34] used transfer learning to train a convolutional neural network (CNN) model on a then expanded dataset of pre- and post-treatment CT scans of 123 bladder cancer patients undergoing neoadjuvant chemotherapy; in another study [35] sarcopenia in metastatic renal carcinoma, according to SMI thresholds after segmentation by the deep learning algorithm, had statistically significant correlation with lower overall survival compared to non-sarcopenic patients [36].

However, to date, there are no studies that have analyzed sarcopenia in advanced urothelial tumors through artificial intelligence software. In this setting, our primary endpoint was to confirm the role of sarcopenia, assessed using a CT based AI-powered software, as a prognostic predictor of objective clinical benefit in terms of tumor response rate to systemic therapy, in advanced urothelial tumors and correlate sarcopenia status with oncological outcomes. As anticipated, we discovered a strong connection between sarcopenia and aging, which is widely recognized as a current health concern among older adults [37,38]. Our results also showed that the number of drugs taken (unrelated to the cancer treatment) and consequently the presence of comorbidities in patients were not statistically significant factors. This finding is in contrast with previous research; Pacifico et al. [39] in a systematic review discovered that individuals with multiple comorbidities, such as cardiovascular diseases, dementia, diabetes [40], and respiratory diseases, had the highest prevalence of sarcopenia [41]. Our outcome is likely a result of the insignificant impact of comorbidities on the sarcopenia status of patients who have advanced cancer.

In this study we found that sarcopenia, assessed by the CT-based AI-powered software is a negative prognostic factor in advanced urothelial cancers; indeed, overall survival was significantly different between the sarcopenic and non-sarcopenic groups. Our data are in line with Yumioka’s and Shimizu’s studies, which demonstrated how sarcopenia is a predictive factor of overall survival in patients affected by urothelial carcinoma and treated with gemcitabine and cis-/carbo-platin [42,43]. Results also confirm recent findings concerning the association of sarcopenia in patients affected by genitourinary tumors and oncologic outcomes [44]. Specifically, sarcopenia has been correlated to a worse prognosis in patients with urothelial carcinoma, including muscle-invasive bladder cancer [45,46] and upper tract urothelial carcinoma by Fukushima et al. in a systematic review of the literature [27]. The findings on genitourinary tumors align with those from systematic reviews and meta-analyses on other types of tumors. It has been shown that pre-treatment sarcopenia is a separate risk factor for both lower overall survival and decreased compliance with adjuvant therapies in pancreatic [47], gastrointestinal [48,49], breast [50], gynecological [51], and hematological [52] cancers. In these prior studies, the presence of sarcopenia was evaluated using commonly accepted methods, such as manually tracing the areas of all muscle groups on CT scans.

It is important to mention that other anthropometric measurements may not be reliable. Indeed, using BMI alone to assess obesity and evaluate outcomes and prognosis in cancer patients is inaccurate since it cannot distinguish between fat and lean mass or between visceral and subcutaneous fat [53].

Although traditional methods of evaluating body composition, such as anthropomorphic measurements, bioelectrical impedance, and dual-energy X-ray absorptiometry, have some drawbacks, computed tomography and magnetic resonance imaging are the benchmark techniques for analyzing body composition. Nevertheless, also these imaging methods demand manual segmentation by an expert reader, which is a time-consuming and labor-intensive task. Consequently, their application in large-scale studies and regular clinical practice is limited [54].

In the present study, we found that integrating a fully automated AI-powered tool into radiological practice, provides an opportunity for innovation to effectively investigate sarcopenia status facilitating the detection of muscle loss and it allows to reduce operator-dependent bias of segmentation in the set of scans routinely acquired for staging and follow-up purposes.

The ability of AI models to analyze large sets of data and extract high-level abstractions beyond manual skills provides impactful information and greatly refines the standard for assessing the risk of muscle depletion in patients with urogenital cancers [55,56] as well as in non-oncological and pediatric patients [57].

This study has several limitations, including its retrospective design and small sample size. Another limitation is the lack of correlation with the patient’s nutritional status. Furthermore, this study, like the other retrospective studies previously described, was not designed to show whether sarcopenia is treatable.

Even though our work is focused on advanced-stage urothelial tumors treated with platinum-based systemic chemotherapy, it would be interesting to evaluate how body composition affects other subtypes of urogenital tumors treated with different therapeutic regimens. Finally, the AI tool’s performance was not compared to manual muscle area segmentation, as the accuracy of the method has already been established in other studies [58].

Despite its limitations, this study marks the first use of CT AI-based body composition measurement in patients with advanced urothelial tumors. Our findings, supported by further evidence, could lead to the development of standardized pathways that link the radiological staging of cancers with sarcopenia assessment and personalized nutritional therapy. This has the potential to enhance both quality of life and cancer outcomes.

## 5. Conclusions

The utilization of an easy-to-use CT-based AI tool allowed us to assess the sarcopenia status of patients with advanced urothelial tumors. Indeed, routine CT scans represented an important imaging biomarker on body composition status, which correlated with poorer oncological outcomes. The AI tool can represent a means to increase the clinical value of CT imaging reports and to promote the implementation of precision medicine [59].

## Figures and Tables

**Table 1:** Patients’ population demographic characteristics and disease outcome according to the body composition status at baseline and after systemic chemotherapy.

Variables	No Sarcopenic Status, Baseline (By SMI-L3 Cut-Off)	Sarcopenic Status, Baseline (By SMI-L3 Cut-Off)	*p*-Value *	No SARCOPENIC Status, after CHT (By SMI-L3 Cut-Off)	Sarcopenic Status, after CHT (By SMI-L3 Cut-Off)	*p*-Value *
**Sample size**, n (%)	46 (47.4)	51 (52.6)		45 (46.4)	52 (53.6)	
	Demographics and tumor-related features					
**Age** y, median (IQR)	73 (64–76)	69 (64–74)	0.414	73 (66–76)	68 (64–74)	0.126
**Age** y, n (%)						
<70 y	19 (41.3)	30 (58.8)	0.105	17 (37.8)	32 (61.5)	**0.025**
≥70 y	27 (58.7)	21 (42.2)		28 (62.2)	20 (38.5)	
**Gender**, n (%)						
Male	36 (78.3)	34 (66.7)	0.259	11 (24.4)	16 (30.8)	0.506
Female	10 (27.7)	17 (33.3)		34 (75.6)	36 (69.2)	
**ECOG PS**, n (%)						
<2	39 (84.8)	40 (78.4)	0.447	38 (84.4)	41 (78.8)	0.603
≥2	7 (15.2)	11 (21.6)		7 (15.6)	11 (21.2)	
**n. of Medications**, n (%)						
<6	36 (78.3)	42 (82.4)	0.620	34 (75.6)	44 (84.6)	0.311
≥6	10 (21.7)	9 (17.6)		11 (24.4)	8 (15.4)	
**Primary location** n (%)						
BCa	24 (52.2)	37 (72.5)	**0.049**	24 (53.3)	37 (71.2)	0.096
UTUC	21 (45.7)	12 (23.5)		20 (44.4)	13 (25.0)	
Concomitant	1 (2.2)	2 (3.9)		1 (2.2)	2 (3.8)	
**Oncologic stage**, n (%)						
III	22 (47.8)	17 (33.3)	0.155	22 (48.9)	17 (32.7)	0.146
IV	24 (52.2)	34 (66.7)		23 (51.1)	35 (67.3)	
	Anthropometric measures					
**Height**, m	1.70 (1.66–1.75)	1.70 (1.62–1.75)	0.753	1.70 (1.64–1.73)	1.70 (1.64–1.75)	0.677
**Weight**, kg	77 (70–85.25)	70 (60–75)	**0.001**	77 (70–80.25)	70 (60–80)	**0.005**
**BMI**, kg/m^2^	26.3 (24.85–27.75)	24.2 (21.9–26.5)	**0.001**	26.3 (25.3–29.3)	23.5 (21.7–26.4)	**0.007**
**SMA**, cm^2^	179.7 (167.8–194)	135.8 (114.8–155.2)	**<0.0001**	173.1 (157.2–193)	133.7 (116–154.4)	**<0.0001**
**SMI-L3**, (cm^2^/m^2^)	62 (57.8–67.1)	48.6 (43.1–53)	**<0.0001**	59.9 (57.3–63.8)	47.9 (43.4–50.9)	**<0.0001**
**Subcutaneous fat**, (cm^2^/m^2^)	184.2 (133.6–221.8)	135.2 (108.4–178.6)	**0.003**	178.7 (130.7–215.1)	143.1 (108.4–183.2)	**0.002**
**Visceral fat**, (cm^2^/m^2^)	207 (153.7–255.7)	103 (67.9–175.7)	**<0.0001**	182.6 (140–218.4)	137 (64.5–181.6)	**<0.0001**
**Psoas muscle**, (cm^2^/m^2^)	21.7 (19.4–24.6)	16.5 (13.3–20)	**<0.0001**	20.8 (18–22.8)	16.1 (13.5–19)	**<0.0001**
**Abdominal muscle**, (cm^2^/m^2^)	100.6 (89.6–109.2)	71.5 (59.1–82.2)	**<0.0001**	93.4 (86.6–106.9)	71.6 (59.7–80.9)	**<0.0001**
**Long spine muscle**, (cm^2^/m^2^)	60.3 (54.8–64.1)	45.5 (40.8–55)	**<0.0001**	57.9 (51.4–62.8)	46.4 (40.7–55.2)	**<0.0001**
**∆_SMI-L3**, mean (SD)	−1.86 (5.78)					
**∆_Subcutaneous fat** mean (SD)	0.25 (24.93)					
**∆_Visceral fat**, mean (SD)	−4.93 (42.17)					
**∆_ Psoas muscle**, mean (SD)	−0.85 (2.56)					
**∆_ Abdominal muscle**, mean (SD)	−3.02 (10.74)					
**∆_ Long spine muscle**, mean (SD)	−1.06 (3.39)					
	Clinical outcomes					
**Clinical Benefit**, n (%)						
SD/PR/CR	30 (65.2)	25 (49)	0.151	29 (64.4)	26 (50.0)	0.217
PD	16 (34.8)	26 (51)		16 (35.6)	26 (50.0)	
**Survival** n (%)						
Deceased	37 (80.4)	23 (45.1)	**0.001**	35 (77.8)	25 (48.1)	**0.003**
Survivors	9 (19.6)	28 (54.9)		10 (22.2)	27 (51.9)	

* *p*-values according to Fisher’s Exact test or Mann-Whitney U test when appropriate (bold *p*-value means that it is statistically significant). CHT, chemotherapy; PS, performance status; BCa, bladder cancer; UTUC, Upper tract Urothelial Carcinoma; BMI, body mass index; SMA, skeletal muscle area; SMI, skeletal muscle mass index; SD, stable disease; PR, partial response; CR, complete response; PD, progression disease.

## Data Availability

The data presented in this study are available upon request from the corresponding author. The data are not publicly available due to restrictions.

## Supplementary Materials

The following supporting information can be downloaded at: https://www.mdpi.com/article/10.3390/cancers15112968/s1, Figure S1: Univariable linear regression plots depicting AI-based Quantib Body Composition^®^ Skeletal Muscle Index (SMI-L3) and anthropomorphic sarcopenia-related variables at baseline (A). Multivariable linear regression model assessing SMI-L3, clinic-demographic and anthropomorphic sarcopenia-related variables at baseline (B). Figure S2: Univariable linear regression plots depicting AI-based Quantib Body Composition^®^ Skeletal Muscle Index (SMI-L3) and anthropomorphic sarcopenia-related variables after fist therapy cycle (A). Multivariable linear regression model assessing SMI-L3, clinic-demographic and anthropomorphic sarcopenia-related variables after fist therapy cycle (B). Table S1: Bivariable and Multivariable adjusted Cox regression modeling for anthropometric measures assessing the Odds Ratio for overall survival at baseline (A) and post-systemic treatment (B). Table S2: Bivariable and Multivariable adjusted Cox regression modeling for anthropometric measures assessing the hazard for overall survival at baseline (A) and post-systemic treatment (B).
