# Supplementary material for: Standardization of Body Composition Status in Patients with Advanced Urothelial Tumors: The Role of a CT-Based AI-Powered Software for the Assessment of Sarcopenia and Patient Outcome Correlation
Source: Cancers (Basel). 2023 May 29;15(11):2968. doi: 10.3390/cancers15112968 (PMC10251974; doi:10.3390/cancers15112968)
Supplement: Supplementary file 1 [file cancers-15-02968-s001.zip › cancers-2320103-supplementary.pdf]

Supplementary Figures

Figure S1 A – S1 B.

Univariable linear regression plots depicting AI-based Quantib Body Composition® Skeletal Muscle Index (SMI-L3) and anthropomorphic sarcopenia-related variables at baseline (A).

Multivariable linear regression model assessing SMI-L3, clinic-demographic and anthropomorphic sarcopenia-related variables at baseline (B).

A

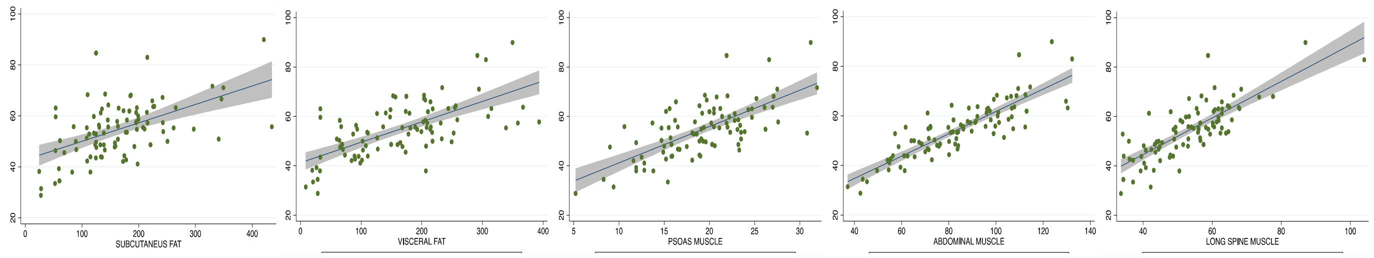

B

Source	Value	Standard error	t	Pr > |t|	Lower bound (95%)	Upper bound (95%)
Intercept	4,258	2,814	1,513	0,134	-1,336	9,851
**Subcutaneous fat**, (cm^2/m^2)	-0,005	0,008	-0,602	0,549	-0,020	0,011
**Visceral fat**, (cm^2/m^2)	0,017	0,007	2,426	0,017	0,003	0,032
**Psoas muscle**, (cm^2/m^2)	0,432	0,131	3,304	0,001	0,172	0,692
**Abdominal muscle**, (cm^2/m^2)	0,304	0,036	8,497	<0,0001	0,233	0,375
**Long spine muscle**, (cm^2/m^2)	0,264	0,060	4,398	<0,0001	0,145	0,384
**Stage disease** (III vs. IV)	-0,825	1,008	-0,819	0,415	-2,829	1,178
**Age, yr** (≤ 70 vs. > 70)	-1,797	0,985	-1,824	0,072	-3,755	0,162
**Gender** (Male vs. Female)	6,644	1,296	5,127	<0,0001	4,068	9,220
**ECOG** (≤1 vs. >1)	-0,384	1,301	-0,295	0,769	-2,970	2,202

**Sum of weights**	97
**DF**	87
R^2	0,857
**Adjusted** R^2	0,843

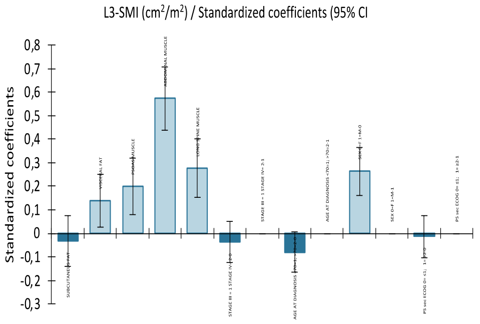

Figure S2 A - B.

Univariable linear regression plots depicting AI-based Quantib Body Composition® Skeletal Muscle Index (SMI-L3) and anthropomorphic sarcopenia-related variables after fist therapy cycle (A).

Multivariable linear regression model assessing SMI-L3, clinic-demographic and anthropomorphic sarcopenia-related variables after fist therapy cycle (B).

A

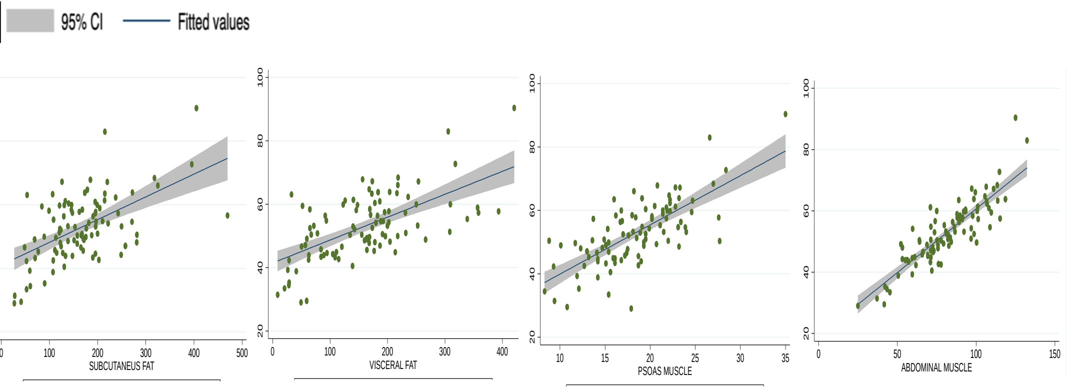

B

Source	Value	Standard error	t	Pr > |t|	Lower bound (95%)	Upper bound (95%)
Intercept	8,419	2,474	3,403	0,001	3,502	13,336
**Subcutaneous fat**, (cm^2/m^2)	0,008	0,007	1,185	0,239	-0,005	0,021
**Visceral fat**, (cm^2/m^2)	0,009	0,006	1,491	0,140	-0,003	0,021
**Psoas muscle**, (cm^2/m^2)	0,377	0,133	2,825	0,006	0,112	0,642
**Abdominal muscle**, (cm^2/m^2)	0,267	0,030	8,893	<0,0001	0,207	0,326
**Long spine muscle**, (cm^2/m^2)	0,252	0,052	4,809	<0,0001	0,148	0,356
**Stage disease** (III vs. IV)	-0,498	0,866	-0,575	0,567	-2,219	1,223
**Age, yr** (≤ 70 vs. > 70)	-1,875	0,823	-2,279	0,025	-3,510	-0,240
**Gender** (Male vs. Female)	4,245	1,164	3,646	0,000	1,931	6,560
**ECOG** (≤1 vs. >1)	-0,236	1,099	-0,214	0,831	-2,420	1,948

**Sum of weights**	97
**DF**	87
**R^2**	0,874
**Adjusted R^2**	0,861

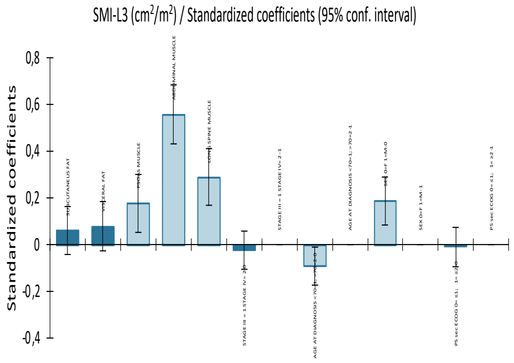

Table S1 A-B

Bivariable and Multivariable adjusted Cox regression modeling for anthropometric measures assessing the Odds Ratio for overall survival at baseline (A) and post-systemic treatment (B).

aOR: adjusted hazard ratio; CI: confidence interval; SMI: skeletal muscle index; Q: quartile; ECOG: Eastern Cooperative Oncology Group

A		Bivariable analysis*		Multivariable analysis**	
Continuous and categorical variables		aOR (95%CI)	P value	aOR (95%CI)	P value
**SMI-L3**, (cm^2/m^2)	Continuous	0.96 (0.92 – 1.1)	0.058	0.95 (0.91 – 0.99)	**0.047**
**Sarcopenic status**, by SMI-L3	No	Ref.		Ref.	
	Yes	2.33 (0.99 – 5.52)	0.054	2.63 (1.05 – 6.61)	**0.039**
**Subcutaneous fat**, (cm^2/m^2)	Continuous	0.99 (0.98 – 0.99)	**0.027**	0.99 (0.98 – 0.99)	**0.023**
**Subcutaneous fat**, (cm^2/m^2)	> Q1	Ref.		Ref.	
	≤ Q1	2.64 (1.07 – 6.92)	**0.048**	2.84 (1.02 – 7.86)	**0.044**
**Visceral fat**, (cm^2/m^2)	Continuous	0.99 (0.99 – 1.00)	0.110	0.99 (0.98 – 1.00)	0.079
**Visceral fat**, (cm^2/m^2)	> Q1	Ref.		Ref.	
	≤ Q1	2.71 (0.92 – 7.93)	0.068	2.91 (0.97 – 8.72)	0.056
**Psoas muscle**, (cm^2/m^2)	Continuous	0.90 (0.82 – 0.99)	**0.026**	0.90 (0.81 – 0.99)	**0.040**
**Psoas muscle**, (cm^2/m^2)	> Q1	Ref.		Ref.	
	≤ Q1	3.09 (0.91 – 10.51)	0.070	2.95 (0.97 – 8.95)	0.055
**Abdominal muscle**, (cm^2/m^2)	Continuous	0.97 (0.95 – 1.00)	**0.021**	0.96 (0.94 – 0.99)	**0.015**
**Abdominal muscle**, (cm^2/m^2)	> Q1	Ref.		Ref.	
	≤ Q1	3.90 (1.01 – 15.05)	**0.048**	3.44 (0.85 – 13.99)	0.084
**Long spine muscle**, (cm^2/m^2)	Continuous	0.97 (0.93 – 1.01)	0.094	0.97 (0.92 – 1.01)	0.117
**Long spine muscle**, (cm^2/m^2)	> Q1	Ref.		Ref.	
	≤ Q1	2.27 (0.77 – 6.70)	0.137	1.98 (0.68 - 5.74)	0.211

*Adjusted by Gender

**Adjusted by Age, Gender, Stage, ECOG, n. of meds

B		Bivariable analysis*		Multivariable analysis**	
**Continuous and categorical variables**		aOR (95%CI)	P value	aOR (95%CI)	P value
**SMI-L3**, (cm^2/m^2)	Continuous	0.93 (0.89 – 0.98)	**0.011**	0.92 (0.87 – 0.97)	**0.006**
**Sarcopenic status**, by SMI-L3	No	Ref.		Ref.	
	Yes	2.00 (0.86 – 4.68)	0.106	2.31 (1.15 – 5.78)	**0.038**
**Subcutaneous fat**, (cm^2/m^2)	Continuous	0.99 (0.98 – 0.99)	**0.041**	0.99 (0.98 – 0.99)	**0.028**
**Subcutaneous fat**, (cm^2/m^2)	> Q1	Ref.		Ref.	0.214
	≤ Q1	1.87 (0.71 – 4.90)	0.199	1.87 (0.69 – 5.07)	
**Visceral fat**, (cm^2/m^2)	Continuous	0.99 (0.99 – 1.00)	0.080	0.99 (0.98 – 1.00)	0.066
**Visceral fat**, (cm^2/m^2)	> Q1	Ref.		Ref.	
	≤ Q1	2.71 (0.92 – 7.93)	0.068	2.77 (0.89 – 8.54)	0.076
**Psoas muscle**, (cm^2/m^2)	Continuous	0.84 (0.74 – 0.94)	**0.005**	0.84 (0.74 – 0.96)	**0.009**
**Psoas muscle**, (cm^2/m^2)	> Q1	Ref.		Ref.	
	≤ Q1	3.09 (0.91 – 10.51)	0.070	2.84 (0.80 – 10.1)	0.106
**Abdominal muscle**, (cm^2/m^2)	Continuous	0.96 (0.93 – 0.99)	**0.007**	0.95 (0.93 – 0.98)	**0.005**
**Abdominal muscle**, (cm^2/m^2)	> Q1	Ref.		Ref.	
	≤ Q1	3.90 (1.01 – 15.05)	**0.048**	3.72 (0.92 – 15.01)	0.064
**Long spine muscle**, (cm^2/m^2)	Continuous	0.95 (0.91 – 0.99)	0.050	0.95 (0.91 – 1.00)	0.053
**Long spine muscle**, (cm^2/m^2)	> Q1	Ref.		Ref.	
	≤ Q1	2.27 (0.77 – 6.70)	0.137	2.67 (0.85 – 8.34)	0.090

*Adjusted by Gender
**Adjusted by Age, Gender, Stage, ECOG, n. of meds

Tables S2 A-B Bivariable and Multivariable adjusted Cox regression modeling for anthropometric measures assessing the hazard for overall survival at baseline (A) and post-systemic treatment (B).

aHR: adjusted hazard ratio; CI: confidence interval; SMI: skeletal muscle index; Q: quartile; ECOG: Eastern Cooperative Oncology Group

A		**Bivariable analysis***		**Multivariable analysis****	
**Continuous and categorical variables**		aHR (95%CI)	P value	aHR (95%CI)	P value
**SMI-L3**, (cm^2/m^2)	Continuous	0.96 (0.92 – 0.98)	**0.005**	0.95 (0.92 – 0.99)	**0.007**
**Sarcopenic status**, by SMI-L3	No	Ref.			
	Yes	3.31 (1.56 – 7.04)	**0.002**	3.79 (1.71 - 8.40)	**0.001**
**Subcutaneous fat**, (cm^2/m^2)	Continuous	1.02 (0.96 – 1.11)	0.169	0.99 (0.99 - 1.001)	0.164
**Subcutaneous fat**, (cm^2/m^2)	> Q1	Ref.		Ref.	
	≤ Q1	1.29 (0.60 – 2.78)	0.506	1.26 (0.58 – 2.73)	0.551
**Visceral fat**, (cm^2/m^2)	Continuous	0,99 (0,98 - 1,02)	0.053	0.99 (0.99 – 1.00)	0.072
**Visceral fat**, (cm^2/m^2)	> Q1	Ref.		Ref.	
	≤ Q1	1.84 (0.89 – 3.79)	0.098	1.74 (0.82 – 3.69)	0.146
**Psoas muscle**, (cm^2/m^2)	Continuous	0.91 (0.85 – 0.97)	**0.007**	0.90 (0.84 – 0.97)	**0.010**
**Psoas muscle**, (cm^2/m^2)	> Q1	Ref.		Ref.	
	≤ Q1	2.03 (1.01 – 4.08)	**0.047**	1.98 (1.16 – 4.08)	**0.049**
**Abdominal muscle**, (cm^2/m^2)	Continuous	0.98 (0.98 – 0.99)	**0.023**	0.98 (0.96 – 0.99)	**0.031**
**Abdominal muscle**, (cm^2/m^2)	> Q1	Ref.		Ref.	
	≤ Q1	2.10 (0.95 – 4.62)	0.064	2.01 (0.88 – 4.58)	0.096
**Long spine muscle**, (cm^2/m^2)	Continuous	0.96 (0.93 – 1.00)	0.053	0.96 (0.93 – 1.00)	0.068
**Long spine muscle**, (cm^2/m^2)	> Q1	Ref.		Ref.	
	≤ Q1	1.68 (0.81 – 3.49)	0.160	1.68 (0.79 – 3.57)	0.175

*Adjusted by Gender
**Adjusted by Age, Gender, Stage, ECOG, n. of meds

B		Bivariable analysis		Multivariable analysis*	
**Continuous and categorical variables**		aHR (95%CI)	P value	aHR (95%CI)	P value
**SMI-L3**, (cm2/m^2)	Continuous	0.95 (0.91 – 0.98)	**0.008**	0.94 (0.91 – 0.98)	**0.009**
**Sarcopenic status**, by SMI-L3	no	Ref.		Ref.	
	yes	2.81 (1.35 – 5.83)	**0.005**	3.29 (1.51 – 7.16)	**0.003**
**Subcutaneous fat**, (cm^2/m^2)	Continuous	0.99 (0.41 – 1.001)	0.585	0.99 (0.99 – 1.001)	0.171
**Subcutaneous fat**, (cm^2/m^2)	> Q1	Ref.		Ref.	
	≤ Q1	1.95 (0.98 – 3.88)		2.02 (1.00 – 4.06)	**0.048**
**Visceral fat**, (cm^2/m^2)	Continuous	0.99 (0.99 – 1.001)	0.080	0.99 (0.99 – 1.001)	0.221
**Visceral fat**, (cm^2/m^2)	> Q1	Ref.		Ref.	
	≤ Q1	1.34 (0.62 – 2.91)	0.445	1.23 (0.55 – 2.74)	0.598
**Psoas muscle**, (cm^2/m^2)	Continuous	0.91 (0.84 – 0.99)	**0.043**	0.90 (0.83 – 0.98)	**0.046**
**Psoas muscle**, (cm^2/m^2)	> Q1	Ref.		Ref.	
	≤ Q1	1.20 (0.50 – 2.83)	0.675	1.14 (0.47 – 2.75)	0.768
**Abd. muscle**, (cm^2/m^2)	Continuous	0.97 (0.96 – 0.99)	**0.028**	0.97 (0.96 – 0.99)	**0.032**
**Abd. muscle**, (cm^2/m^2)	> Q1	Ref.		Ref.	
	≤ Q1	1.97 (0.87 – 4.45)	0.100	1.89 (0.82 – 4.39)	0.133
**Long spine muscle**, (cm^2/m^2)	Continuous	0.97 (0.93 – 1.00)	0.135	0.97 (0.93 – 1.001)	0.154
**Long spine muscle**, (cm^2/m^2)	> Q1	Ref.		Ref.	
	≤ Q1	1.10 (0.49 – 2.49)	0.803	1.13 (0.49 – 2.57)	0.768

*Adjusted by Gender
**Adjusted by Age, Gender, Stage, ECOG, n. of meds
